# Author Correction: Commensal microflora-induced T cell responses mediate progressive neurodegeneration in glaucoma

**DOI:** 10.1038/s41467-018-06428-2

**Published:** 2018-09-20

**Authors:** Huihui Chen, Kin-Sang Cho, T. H. Khanh Vu, Ching-Hung Shen, Mandeep Kaur, Guochun Chen, Rose Mathew, M. Lisa McHam, Ahad Fazelat, Kameran Lashkari, Ngan Pan Bennett Au, Joyce Ka Yu Tse, Yingqian Li, Honghua Yu, Lanbo Yang, Joan Stein-Streilein, Chi Him Eddie Ma, Clifford J. Woolf, Mark T. Whary, Martine J. Jager, James G. Fox, Jianzhu Chen, Dong F. Chen

**Affiliations:** 1 Department of Ophthalmology, Second Xiangya Hospital of Central South University Changsha, Hunan Province, 410011 Hunan China; 2 Institution of Ophthalmic Center, Changsha, Hunan Province, 410011 Hunan China; 3 Schepens Eye Research Institute of Massachusetts Eye and Ear, Harvard Medical School, Boston, 02114 MA USA; 4 Department of Ophthalmology, Harvard Medical School, Boston, 02114 MA USA; 5 Department of Ophthalmology, Leiden University Medical Center, 2333 ZA Leiden, The Netherlands; 6 Koch Institute for Integrative Cancer Research, Massachusetts Institute of Technology, Cambridge, 02139 MA USA; 7 Department of Biology, Massachusetts Institute of Technology, Cambridge, 02139 MA USA; 8 Department of Nephrology, Second Xiangya Hospital of Central South University, Changsha, 410011 Hunan China; 9 Massachusetts Eye Health Service, Boston, 02124 MA USA; 10 Department of Biomedical Sciences, City University of Hong Kong, Tat Chee Avenue, Hong Kong, China; 11 F.M. Kirby Neurobiology Center, Children’s Hospital Boston, Harvard Medical School, Boston, 02115 MA USA; 12 Department of Neurobiology, Harvard Medical School, Boston, 02115 MA USA; 13 Department of Biological Engineering, Massachusetts Institute of Technology, Cambridge, 02139 MA USA; 14 Division of Comparative Medicine, Massachusetts Institute of Technology, Cambridge, 02139 MA USA

Correction to: *Nature Communications*; 10.1038/s41467-018-05681-9; published online 10 August 2018

The originally published version of this Article contained an error in Fig. 4. The bar chart in panel f was inadvertently replaced with a duplicate of the bar chart in panel e. This error has now been corrected in both the PDF and HTML versions of the Article.

