# Supplementary material for: Commensal microflora-induced T cell responses mediate progressive neurodegeneration in glaucoma
Source: Nat Commun. 2018 Aug 10;9:3209. doi: 10.1038/s41467-018-05681-9 (PMC6086830; doi:10.1038/s41467-018-05681-9)
Supplement: Supplementary file 1 — Supplementary Information [file 41467_2018_5681_MOESM1_ESM.pdf]

Supplementary Information

Commensal Microflora-induced T Cell Responses Mediate Progressive Neurodegeneration in Glaucoma

Chen et al.

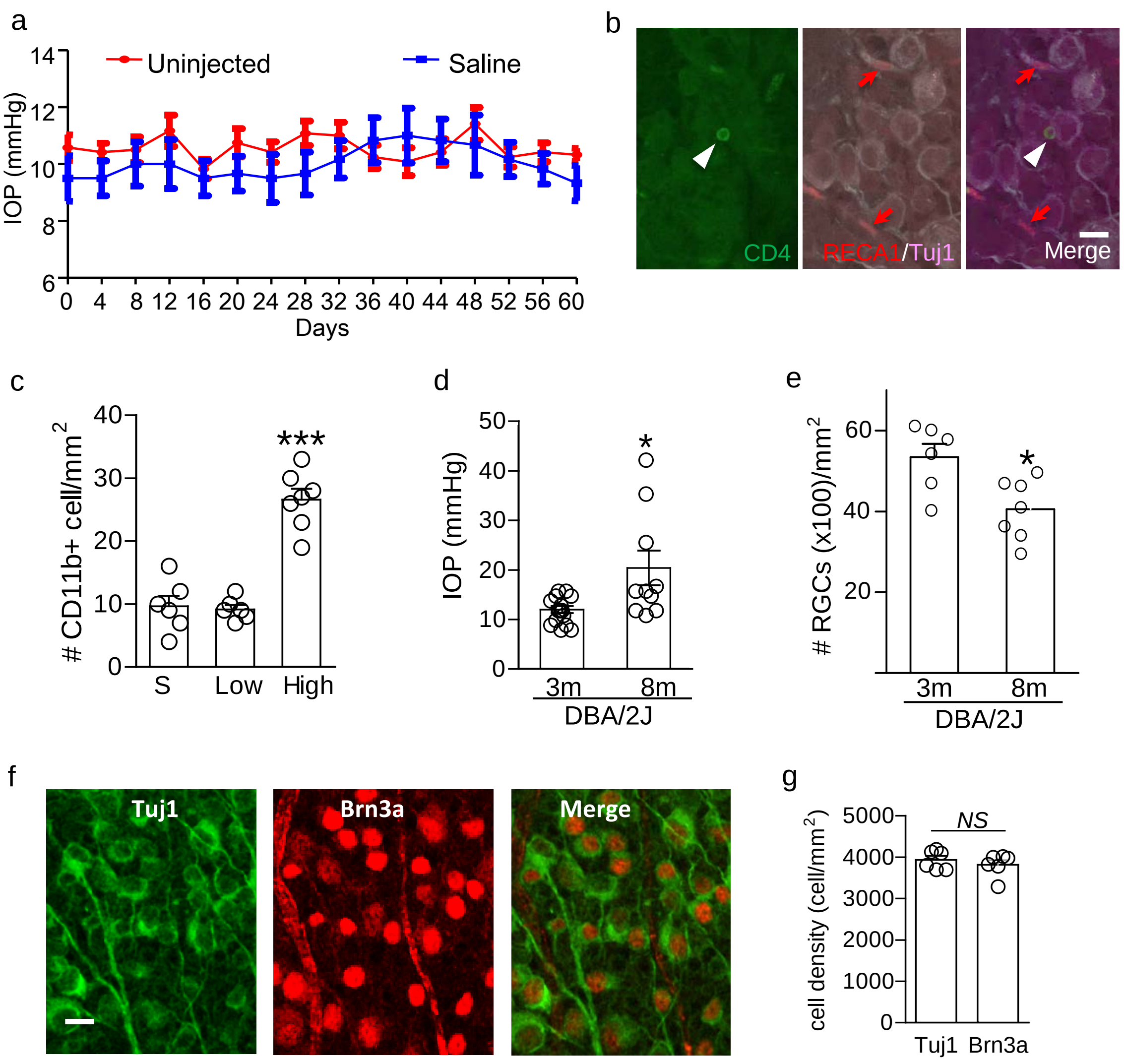

Supplementary Fig. 1. Comparison of IOP kinetics and quantification of CD11b$^+$ cells and infiltrated T cells in saline- and MB-injected mice. (**a**) IOP levels in saline-injected (blue) or un-injected (red) mouse eyes. (**b**) Representative images taken from a retinal of a MB-injected eye, which was triple-stained with antibodies for CD4 (green), RECA1 (red) and Tuj1 (purple). Arrowhead points to a CD4$^+$ T cell; arrows point to RECA1$^+$ blood vessel cells. Scale bar: 10μm. (**c**) Quantification of CD11b$^+$ cells in the retinas of mice 2 weeks after saline, low- (Low; 2.0×10^6 beads/eye) or high- (High; 5.0×10^6 beads/eye) dose injection of MB. ****P*<0.001 as compared to saline injected group (n=6/group). (**d,e**) Quantification of IOP levels (**d**) and RGC densities (**e**) in 3 (n=6) and 8 (n=7) month old DBA/2J mice. **P*<0.05 by ANOVA. (**f,g**) Representative images of retinal flat-mount double-immunolabeled with Tuj1 (green) and anti-Brn3a (red) (**f**) and quantification of Tuj1$^+$ and Brn3a$^+$ cell density in B6 mice (**g**). Scale bar: 10μm. NS: *P*>0.05 by ANOVA (n=6/group).

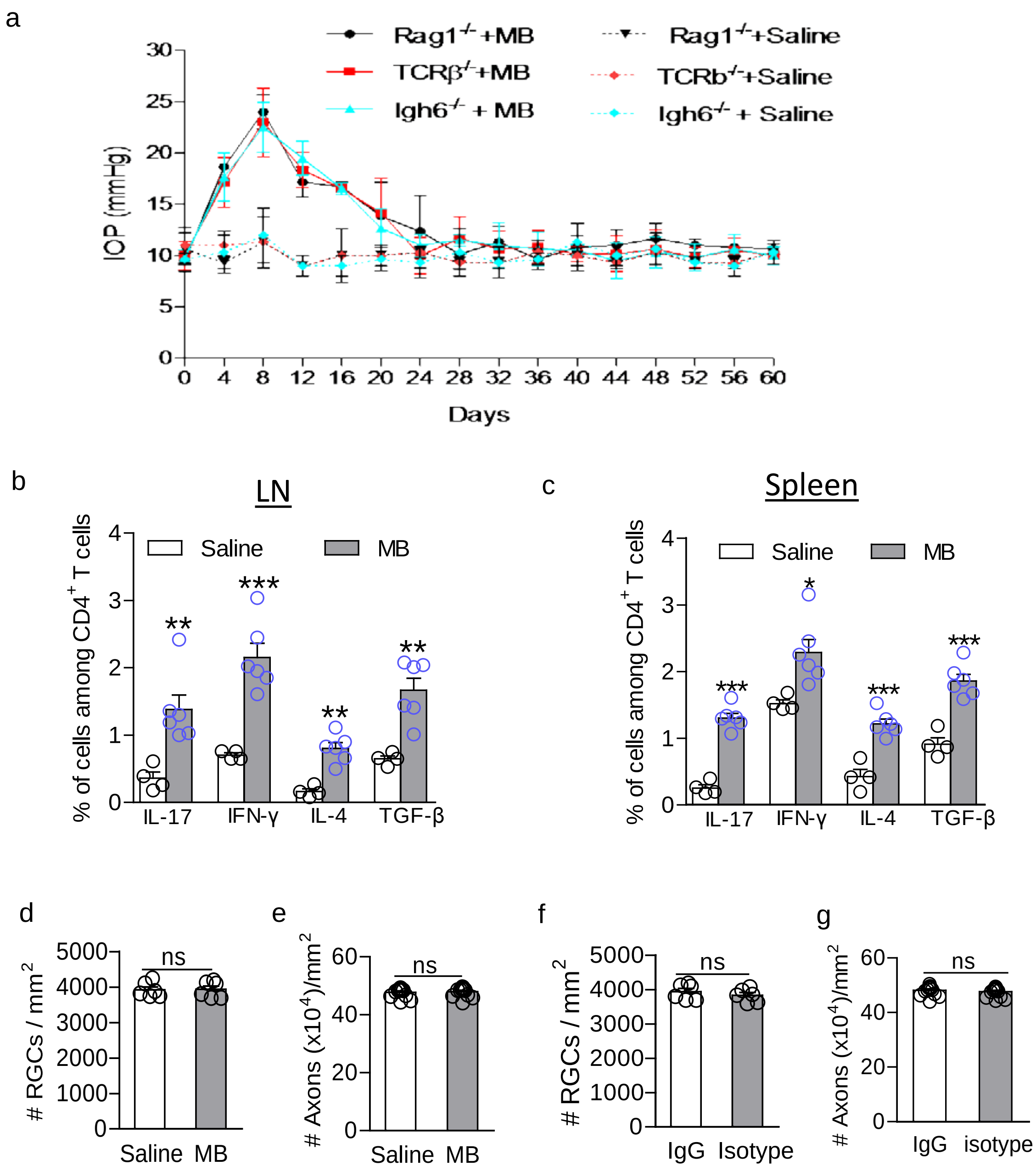

a
Rag1-/- +MB
TCRβ-/- +MB
Igh6-/- + MB
Rag1-/- +Saline
TCRb-/- +Saline
Igh6-/- + Saline
IOP (mmHg)
0
5
10
15
20
25
30
0 4 8 12 16 20 24 28 32 36 40 44 48 52 56 60
Days
b
LN
Saline
MB
% of cells among CD4+ T cells
0
1
2
3
4
**
***
**
**
IL-17
IFN-γ
IL-4
TGF-β
c
Spleen
Saline
MB
% of cells among CD4+ T cells
0
1
2
3
4
***
*
***
***
IL-17
IFN-γ
IL-4
TGF-β
d
RGCs / mm2
0
1000
2000
3000
4000
5000
ns
Saline MB
e
Axons (x104)/mm2
0
20
40
60
ns
Saline MB
f
RGCs / mm2
0
1000
2000
3000
4000
5000
ns
IgG Isotype
g
Axons (x104)/mm2
0
20
40
60
ns
IgG isotype

Supplementary Fig. 2. IOP kinetics in genetic mouse lines, systemic T cell profiling, and RGC and axon counts in retinas of control *Rag1*$^{-/-}$ mice. (**a**) IOP levels over time in *Rag1*$^{-/-}$, *TCRβ*$^{-/-}$ and *Igh6*$^{-/-}$ mice following anterior chamber injection of saline or MB (n≥6/group). (**b,c**) Flow cytometry analysis of systemic CD4^{+} T cell profile in the cervical LN (**b**) and spleen (**c**) of saline- (white bars) and MB-injected (black bars) mice. Mice were sacrificed 2 weeks post injection (n≥4/group). Note the significant increase in frequencies of all 4 subtypes of CD4^{+} T cells expressing IFN-γ, IL-17, IL-4, or TGF-β, in MB injected mice when were compared with control mice. *$P<0.05$, **$P<0.01$, ***$P<0.001$ by ANOVA. (**d,e**) Quantification of RGC (**d**) and axon (**e**) density in the retinas of *Rag1*$^{-/-}$ mice 2 weeks following adoptive transfer of CD4^{+} T cells isolated from saline- (Saline) or MB- (MB) injected B6 mice. $P>0.05$ by ANOVA (n=6/group). (**f,g**) Quantification of RGC (**f**) and axon (**g**) density in *Rag1*$^{-/-}$ mice that received intravitreal injection of isotype (Isotype) or total IgGs isolated from MB-injected B6 mice (IgG). $P>0.05$ by ANOVA (n=6/group).

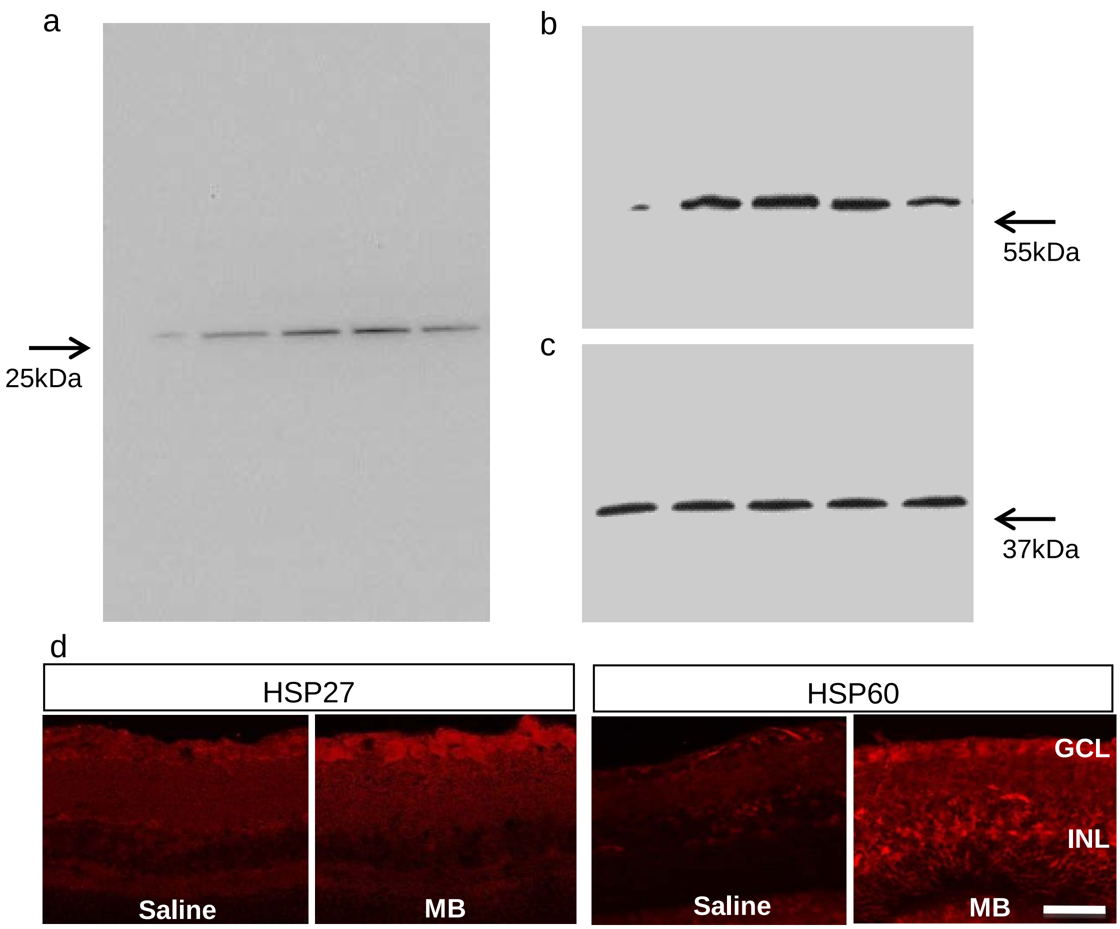

Supplementary Fig. 3. Western blot analysis and immunofluorescence detection of HSP induction following elevated IOP. (**a-c**) Representative images of Western blots detecting HSP27 and HSP60 expression in the retinas of saline and MB injected mice (original uncropped scans of Fig. 3a). The position of the molecular size marker is indicated. (**d**) Photomocrographs of HSP27 and HSP60 immunolabeled retinal sections taken from saline- or MB-injected mice. Note the GCL localization of HSP27 and the wider distribution of HSP60 labeling in MB-injected retinas. Bar: 40 µm.

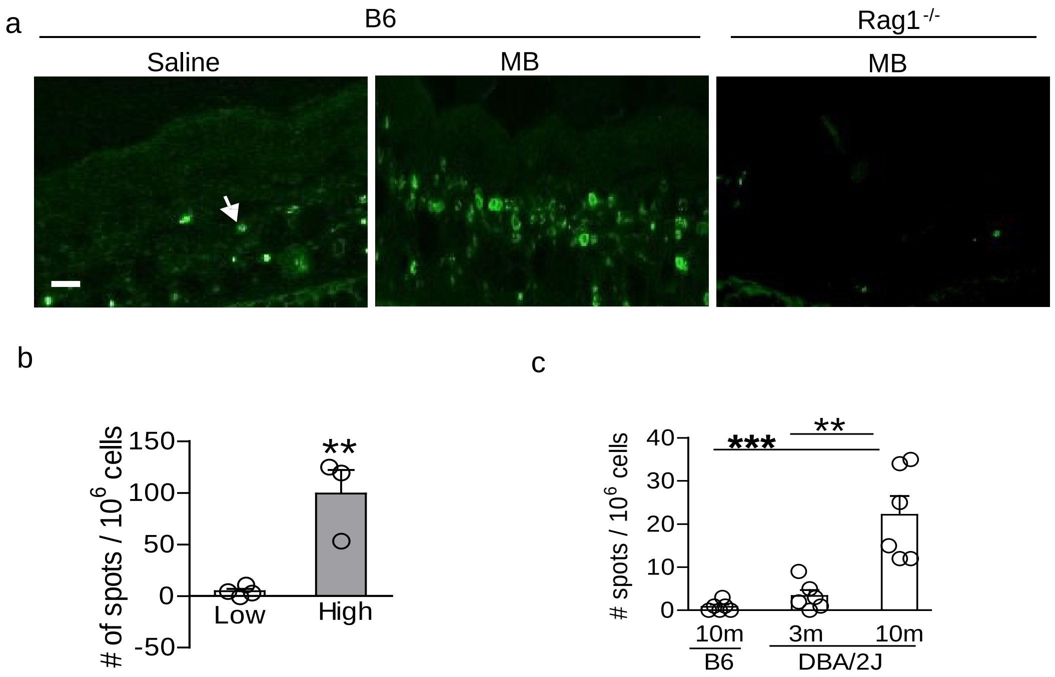

Supplementary Fig. 4. Elevated IOP-induced T cell responses to HSPs. (**a**) Induction of HSP27-specific T cell responses in MB-injected B6 mice, but not in *Rag1*$^{-/-}$ mice. B6 and *Rag1*$^{-/-}$ mice were injected with saline or MB. Two weeks later, mice were challenged with Hsp27 in the ears and sacrificed 24 hours later. Ear sections were immunolabeled with anti-CD4 (green) and representative epifluorescence photomicrographs are shown. (**b**) Comparison of frequencies of HSP27-specific T cells in mice received a low- or high-dose MB injection: Adult B6 mice were injected with a low- (Low; 2.0x10^6) or high- (High; 5.0×10^6) dose of MB in the anterior chamber of the eye. Two weeks later splenocytes were isolated and stimulated with HSP27 in culture; IFN-γ-secreting cells were detected by ELISPOT after 3 days of incubation. ***P*<0.01 by ANOVA (n≥3/group). (**c**) Frequencies of HSP27-specific T cell responses in DBA/2J mice. Splenocytes were harvested from naïve B6 (n=8) and 3 months (n=8) and 8 months old (n=6) DBA/2J mice and assayed for frequencies of IFN-g-secreting cells by ELISPOT following Hsp27 stimulation. Bar: 10µm. ****P*<0.001 by ANOVA.

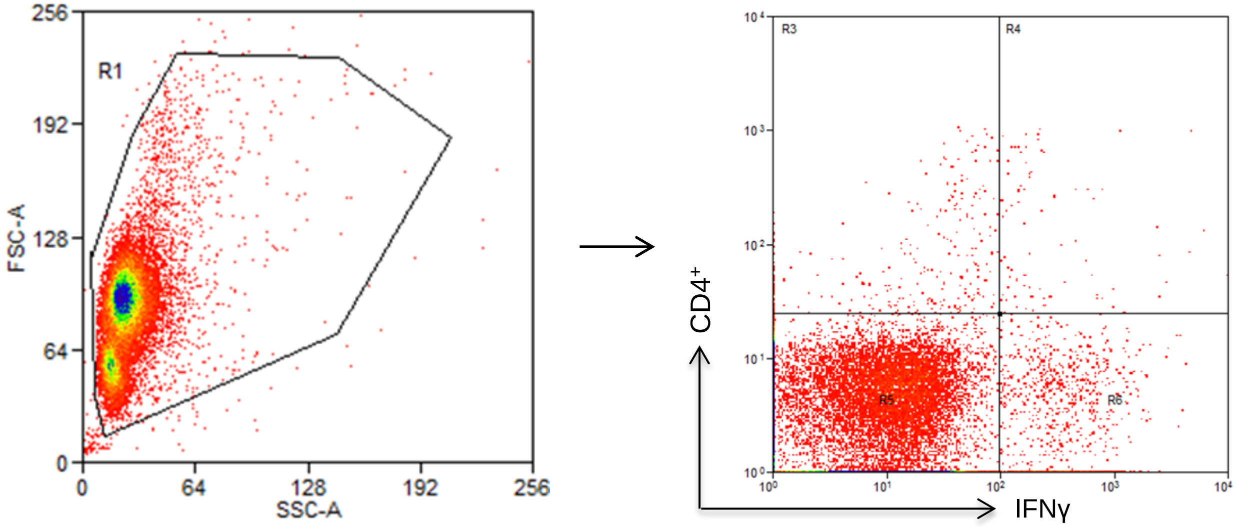

Supplementary Fig. 5. Gating strategy for CD4^{+} T cell quantification. Representative flow cytometry dot plots and the employed gating strategy for the quantification of CD4^{+}/IFNγ^{+} T cells in HSP27-stimulated retinal cells after culturing.

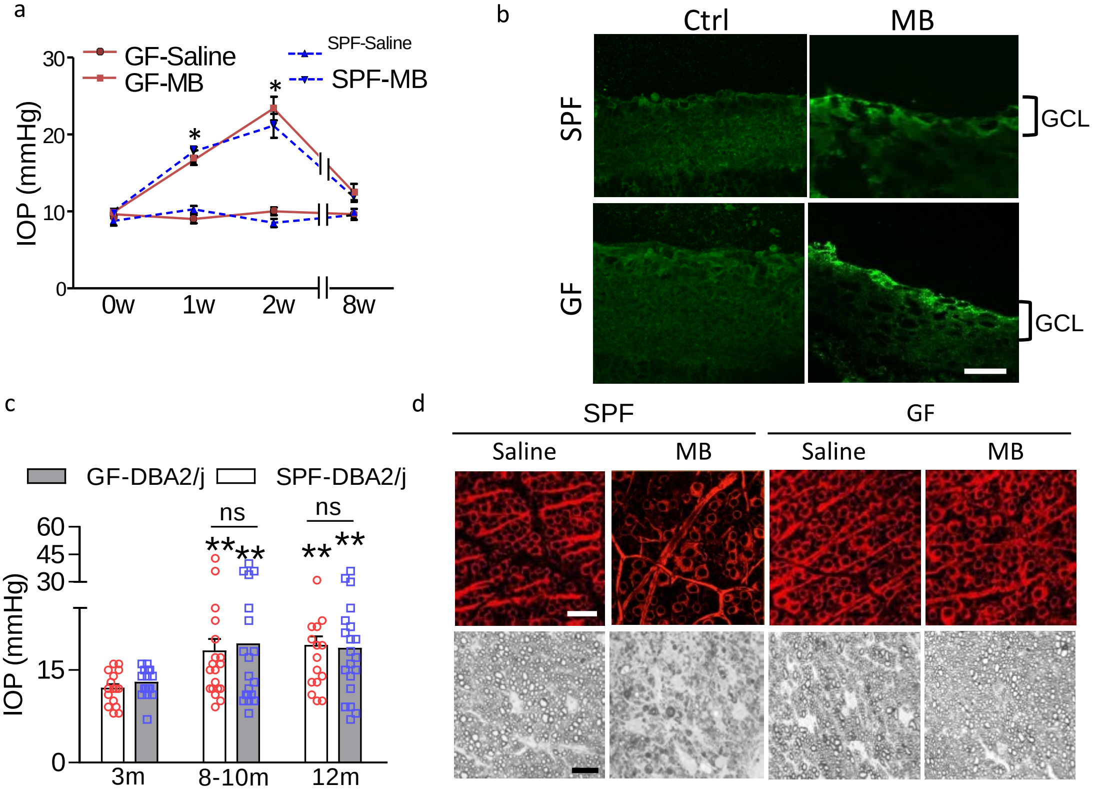

Supplementary Fig. 6. IOP kinetics, T cell infiltration, and RGC degeneration in SPF and GF mice. (**a**) IOP levels in SFP and GF Swiss-Webster mice before (0w) and 1, 2, and 8 weeks after anterior chamber injection of MB or saline. *P<0.001 as compared to saline injected eyes (n≥10/group). (**b**) Up-regulation of HSP27 in the retinas of SPF and GF SW mice at 1 week after MB injection. Retinal sections were stained with anti-HSP27 (green) (n=5/group). Scale bar: 15 µm. (**c**) IOP levels in SPF and GF DBA/2J mice at 3, 8-10, and 12 months of age. **P<0.01 as compared to 3 months old mice by ANOVA (n≥10/group). (**d**) Retinal flat-mounts immunolabeled with Tuj-1 (red) and electron microscopy of optic nerve cross sections from SPF and GF Swiss-Webster mice at 8 weeks after saline or MB injection. Bar: 50µm .
